# The Role of Clothing in the Infection of War Wounds

**Published:** 1918-01

**Authors:** 


***The Rôle of Clothing in the Infection of War Wounds.*** By M. Paul CARNOT, Médecin-major de 1re classe, Medical Chief of the XVe Region. Trans. and Abs. from the *Archives de Médecine et de Pharmacie Militaires*, Aug. 1916, t. LXVI, N° 2, p. 221.

C., in the course of a detailed study of the part played by clothing in wound infection, carried out many experiments to find a means of rendering clothing proof against infectious organisms. The most satisfactory means of doing so, he found to be the use of various substances that render clothing impermeable. To these, various antiseptics may be added. He tried, with success, gelatine, rubber, oils, paraffin, and metallic salts, cresyl, naphthol, and eucalyptus. The antiseptics may be introduced either by proper solvents, or, as in the case of metallic salts, by the deposit on the fibers of the cloth of antiseptic soaps of copper and zinc.

The author has in laboratory experiments obtained excellent results with formol in gelatine, but he remarks that the formol must be frequently renewed.

